# PP2A mediates apoptosis or autophagic cell death in multiple myeloma cell lines

**DOI:** 10.18632/oncotarget.20415

**Published:** 2017-08-23

**Authors:** Hang Zhou, Wei Luo, Chao Zeng, Yu Zhang, Liyang Wang, Wenxiu Yao, Chunlai Nie

**Affiliations:** ^1^ Department of Chemotherapy, Sichuan Cancer Hospital & Institute, Sichuan Cancer Center, School of Medicine, University of Electronic Science and Technology of China, Chengdu, China; ^2^ Department of Pharmacy, Sichuan Cancer Hospital & Institute, Sichuan Cancer Center, School of Medicine, University of Electronic Science and Technology of China, Chengdu, China; ^3^ Department of Gastroenterology, the Third People's Hospital of Chengdu, Chengdu, China; ^4^ Laboratory of Biotherapy and Cancer Center, West China Hospital, Sichuan University and Collaborative Innovation Center for Biotherapy, Chengdu, China; ^5^ Department of Oncology, Guizhou People's Hospital, Guizhou, China

**Keywords:** chemoresistance, autophagy, apoptosis, Beclin-1, PP2A

## Abstract

The crosstalk between apoptosis and autophagy contributes to tumorigenesis and cancer therapy. The process by which BetA (betulinic acid), a naturally occurring triterpenoid, regulates apoptosis and autophagy as a cancer therapy is unclear. In this study, we show for the first time that protein phosphatase 2A (PP2A) acts as a switch to regulate apoptosis and autophagic cell death mediated by BetA. Under normal conditions, caspase-3 is activated by the mitochondrial pathway upon BetA treatment. Activated caspase-3 cleaves the A subunit of PP2A (PP2A/A), resulting in the association of PP2A and Akt. This association inactivates Akt to initiate apoptosis. Overexpression of Bcl-2 attenuates the mitochondrial apoptosis pathway, resulting in caspase-3 inactivation and the dissociation of PP2A and Akt. PP2A isolated from Akt binds with DAPK to induce autophagic cell death. Meanwhile, *in vivo* tumor experiments have demonstrated that BetA initiates different types of cell death in a myeloma xenograft model. Thus, PP2A can shift myeloma cells from apoptosis to autophagic cell death. These findings have important implications for the therapeutic application of BetA, particularly against apoptosis-resistant cancers.

## INTRODUCTION

Multiple myeloma (MM), or plasma cell myeloma, is a malignant accumulation of secretory plasma cells within the bone marrow [1, 2]. Chemotherapies such as lenalidomide and bortezomib have improved survival, but myeloma remains largely incurable [3]. One of the challenges in treating myeloma is chemoresistance [4]. The precise mechanism underlying chemoresistance in multiple myeloma is unclear, but one of the main contributors to both chemoresistance and pathogenesis is thought to be the dysregulation of apoptosis [4, 5]. Overexpression of anti-apoptotic molecules such as Bcl-2 and Bcl-xL has been linked to chemoresistance in myeloma [4, 6].

Bcl-2 overexpression is found in the majority of myeloma patients and plasma cell lines [4, 7, 8]. Bcl-2 protects against apoptosis by neutralizing the function of pro-apoptotic Bcl-2 family members such as Bax and Puma and by preventing cytochrome c release from the mitochondria and subsequent apoptotic events, including caspase-3 activation [9]. Bcl-2 overexpression can also rescue myeloma cells from glucocorticoid-induced apoptosis [10] and confer protection from apoptosis induced by IL-6 deprivation [11]. Moreover, upregulated protein expression of Bcl-2 contributes to the survival of tumor cells treated with either etoposide or doxorubicin (Dox) [12]. Therefore, abrogating or overcoming the anti-apoptotic function of Bcl-2 may increase chemosensitivity and reverse chemoresistance in myeloma tumor cells.

Autophagy, which is characterized as a self-digestive process that ensures lysosomal degradation of long-lived proteins and organelles to maintain cellular homeostasis, has been confirmed as having an indispensable role in tumorigenesis and tumor therapy [13–15]. Autophagy is important for conferring resistance against chemotherapy and targeted therapies [15–17]. A recent study revealed that Beclin-1-mediated autophagy enhances the effectiveness of EGFR tyrosine kinase inhibitor (TKI) therapy in non-small cell lung carcinoma (NSCLC) cells [16]. Conversely, Beclin-1-induced autophagy decreases the sensitization of hepatoma cells to chemotherapeutic agents and mediates the chemoresistance under hypoxia [18]. These results revealed that autophagy exerts dual effects on cancer therapy. Indeed, many studies have demonstrated similar observations [13, 14, 19–23]. On the one hand, as a protective mechanism, autophagy mediates the acquired chemoresistance in some cancer cells. Thus, inhibiting autophagy can restore the effects of chemotherapeutic agents against resistant cancer cells. On the other hand, autophagy may also function as a death initiator to trigger autophagic cell death, a form of physiological cell death which is contradictory to type I programmed cell death (apoptosis) [24]. Therefore, chemotherapeutic induction of autophagic cell death is an effective strategy in treating apoptosis-deficient cancer cells. A previous study demonstrated that autophagy could mediate cell death in HeLa cells overexpressing Bcl-xL and in PUMA- or Bax-deficient human colon cancer cells [20, 22].

PP2A, the main serine/threonine phosphatase in mammalian cells, contributes to a variety of cellular processes, such as mitosis and DNA damage [25, 26]. Several studies have revealed that PP2A could induce apoptosis [27–29]. Recently, PP2A was reported to regulate autophagy in cells [30, 31]. These studies indicated that PP2A may be a key regulator of apoptosis and autophagy. Meanwhile, BetA (betulinic acid) was initially found to induce Bcl-2 family-dependent apoptosis. However, some studies have also shown that BetA could induce Bax/Bak-independent cell death [32, 33]. Eventually, it was revealed that neither Bcl-2 overexpression nor apoptosis inhibition via caspase inhibitors could prevent BetA-induced cell death [34], indicating that BetA could initiate apoptosis-independent cell death.

In this study, we first found that BetA could induce PP2A activation in multiple myeloma cells with differential Bcl-2 expression. We provided evidence that PP2A could mediate Akt-dependent apoptosis in cells with low levels of Bcl-2 expression and DAPK-dependent autophagic cell death in cells with high levels of Bcl-2 expression. Our results demonstrate that PP2A could be used as a switch between apoptosis and autophagic cell death in myeloma to enhance the effects of chemotherapeutic agents.

## RESULTS

### BetA induces apoptosis-independent cell death in cells with high levels of Bcl-2 expression and apoptosis in cells with low levels of Bcl-2 expression

**Figure 1:**
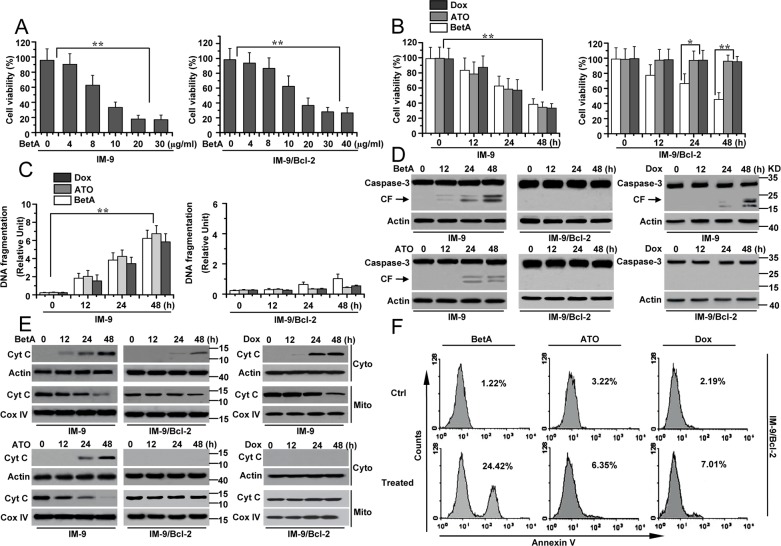
BetA induces apoptosis-independent cell death in IM-9/Bcl-2 and apoptosis in IM-9 cancer cells **(A)** Analysis of cell viability treated with BetA. IM-9 and IM-9/Bcl-2 cells were treated with different concentrations of BetA for 48 h. Cell viability was determined as described in Materials and methods. A minimum of 100 cells per sample for triplicate samples was counted per condition per experiment. Graphs showing results of quantitative analyses (*n*=3, mean ± S.D. **,*P*<0.01, the compared groups: 20 μg/ml & 0; 20 μg/ml & 4μg/ml; 30 μg/ml & 0; 30 μg/ml & 4μg/ml in IM-9 cells; 30 μg/ml & 0; 30 μg/ml & 4μg/ml; 40 μg/ml & 0; 40 μg/ml & 4μg/ml in IM-9/Bcl-2 cells). **(B)** Detection of cell viability. Cells were treated with BetA (10 μg/ml), ATO (3 μM) or Dox (300 nM) for different periods of time. Cell viability was examined as described in panel **A**. Graphs showing results of quantitative analyses (*n*=3, mean ± S.D. **,*P*<0.01, the compared groups: 48 h & 0 h under the same agent treatment in IM-9 cells; Dox or ATO & BetA treatment at 24 h or 48 h in IM-9/Bcl-2 cells). **(C)** Analysis of apoptosis treated with BetA. Cells were treated as described in panel **B**, Apoptotic cell death was quantitatively detected by a cell death ELISA kit as described in Materials and methods. Graphs showing results of quantitative analyses (*n*=3, mean ± S.D. *,*P*<0.05; **,*P*<0.01, the compared groups: 48 h & 0 h under the same agent treatment in IM-9 cells). **(D)** Cells were treated as described in panel **B**. Treated cells were collected for detecting casapse-3 activation. β-Actin was used as a protein loading control. **(E)** As described in panel **B**, treated cells were subjected to subcellular fractionation. The cytosolic and mitochondrial fractions were immunoblotted for detecting the release of Cyt c. β-Actin and Cox IV was used as a protein loading control. **(F)** IM-9/Bcl-2 cells were treated with 10 μg/ml BetA, 3 μM ATO or 300 nM Dox for 48 h, and then cells were collected for Annexin V staining. Representative results of three experiments with consistent results are shown.

We first detected the expression of Bcl-2 in IM-9 and IM-9/Bcl-2 cells (Supplementary Figure 1A). Our results indicated that Bcl-2 was overexpressed in IM-9/Bcl-2 cells. BetA induced a very potent form of cell death in IM-9 and IM-9/Bcl-2 cells (Figure 1A and 1B). The effect of BetA was concentration-dependent as previously described [34], starting at approximately 8 μg/ml in both cell lines and peaking at 20-30 μg/ml in IM-9 cells and 30-40 μg/ml in IM-9/Bcl-2 cells (Figure 1A). The IC_50_ of BetA was approximately 9 μg/ml in IM-9 cells and 10 μg/ml in IM-9/Bcl-2 cells. Thus, we used 10 μg/ml BetA for all subsequent experiments. Meanwhile, Arsenic oxide (ATO), which can induce apoptosis in IM-9 cells but not in IM-9/Bcl-2 cells [35], and Doxorubicin (Dox), which is a topoisomerase inhibitor [36], were used as controls. However, ATO or Dox decreased cell viability in IM-9 cells but not in IM-9/Bcl-2 cells (Figure 1B).

We then determined whether BetA could induce apoptosis in IM-9 and IM-9/Bcl-2 cells. We treated cells with BetA at the indicated times, and apoptosis was assessed by a DNA fragmentation ELISA. As depicted in Figure 1C, BetA efficiently induced apoptosis in IM-9 cells but not in IM-9/Bcl-2 cells. The same results were obtained upon treatment with ATO or Dox. Western blot analysis also revealed that BetA, ATO and Dox could induce caspase-3 cleavage in IM-9 cells but not in IM-9/Bcl-2 cells (Figure 1D). Further experiments demonstrated that BetA could induce the sufficient release of cytochrome C (Cyt c) in IM-9 cells. Meanwhile, we also detected a small amount of Cyt c in the cytosol of IM-9/Bcl-2 cells. However, ATO or Dox could initiate the release of Cyt c in IM-9 cells but not in IM-9/Bcl-2 cells (Figure 1E).

Meanwhile, we also used the 8226 and U-266-1970 myeloma cell lines as models to detect the effect of BetA. 8226 and U-266-1970 cells have low and high levels of Bcl-2 expression, respectively [7, 37] (Supplementary Figure 1B). ATO and Dox could initiate cell apoptosis in 8226 but not U-266-1970 cells, whereas BetA caused apoptosis-independent cell death in U-266-1970 cells (Supplementary Figure 1C and 1D). These results revealed that BetA induces non-apoptotic cell death in cells with high levels of Bcl-2 expression while initiating apoptosis in cells with low levels of Bcl-2 expression.

Interestingly, many annexin V-positive cells were detected in BetA-treated but neither in ATO- nor Dox-treated cells with high levels of Bcl-2 expression (Figure 1F, Supplementary Figure 1E and 1F). Since annexin V staining can detect caspase-independent cell death such as autophagy [38], we speculated that BetA initiates autophagic cell death in cells with high levels of Bcl-2 expression.

### BetA induces autophagic cell death in cells with high levels of Bcl-2 expression

**Figure 2:**
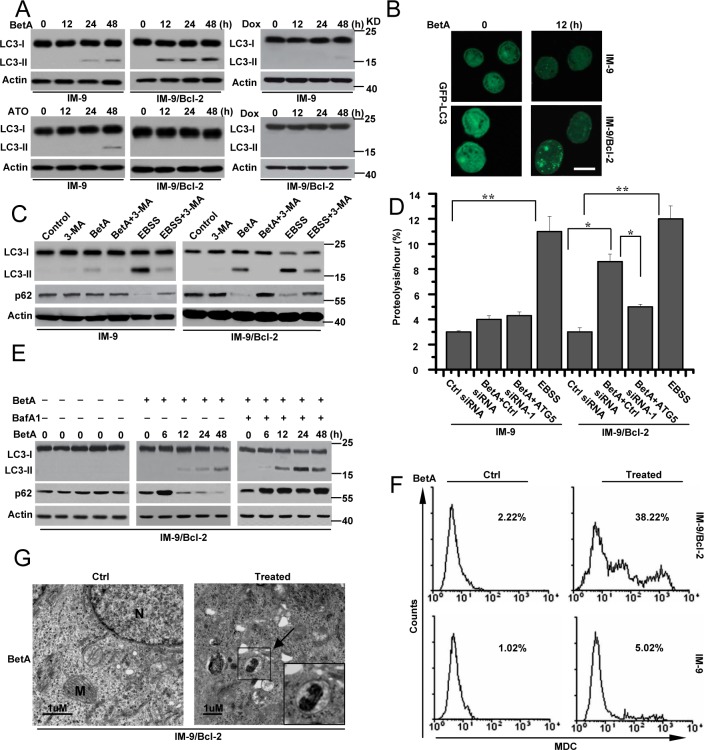
BetA induces autophagic cell death in IM-9/Bcl-2 not in IM-9 cells **(A)** immunoblot analysis of LC3-I and LC3-II levels in cells. Cells were treated as described in Figure 1, and then treated cells were collected for detecting LC3-I and LC3-II content by Western blotting, with β-Actin serving as a loading control. **(B)** Cells were treated as described in **A**. IM-9/Bcl-2/GFP-LC33 cell lines were treated with BetA for 12 h, fixed, and then visualized by fluorescent microscopy, Bars, 10 μM. **(C)** Autophagosome inhibitor 3-MA attenuated the effect of BetA on autophagy in IM-9/Bcl-2 cells. Cells were treated with BetA for 48 h in the absence or presence of the inhibitor of class III PI3 kinases 3-MA (5 mM), lysed and subjected to western blotting with anti-LC3 or p62 antibodies to monitor autophagy. EBSS, (starvation medium) is as a control, which is a classical stimulus used to induce the build-up of autophagosomes and autophagic flux. **(D)** BetA promotes long-lived protein degradation in cells cultured in full medium. IM-9 and IM-9/Bcl-2 cells were first transfected with Ctrl or ATG5 siRNA-1 for 24 h, and then radiolabeled for 24 h with 0.05 mCi/ml of L-[U-^14^C]valine. At the end of the labeling period, the cells were rinsed three times with phosphate-buffered saline. The cells were then incubated in full medium in the presence or in the absence of 10 μg/ml BetA or EBSS with 10 mM valine for 12 h. The data are presented as the means ± S.D. from three independent experiments (*,*P*<0.05; **,*P*<0.01). **(E)** IM-9/Bcl-2 cells were treated with BetA, and then 20 nM of BafA1 was added to the culture medium 2 h before cell lysis. Cells were treated with BetA at indicated time in the absence or presence of BafA1. p62 expression and LC3 conversion were detected by immunoblot analysis, with β-Actin serving as a loading control. **(F)** Indicated cell lines were treated with BetA for 48 h, stained with MDC and analyzed by flow cytometry. **(G)** Representative electron micrographs of IM-9/Bcl-2 treated or untreated with 10 μg/ml BetA for 24 h. *Arrows*, presence of autophagosomes. *N*, nucleus; *M*, mitochondria. Bars, 1μM. All data are representative of three independent experiments.

To confirm our hypotheses, we examined the conversion of the cytoplasmic protein LC3-I protein to its preautophagosomal and autophagosomal membrane–bound form LC3-II, which is a specific marker of autophagosomes in several tested cell lines [39]. As shown in Figure 2A, the immunoblotting analysis revealed that BetA treatment led to a weak time-dependent increase in the levels of LC3-II protein in IM-9 cells and a clear increase of LC3-II expression in IM-9/Bcl-2 cells. Moreover, we found that 8 μg/ml BetA did not efficiently induce the conversion of LC3-I to LC3-II in IM-9/Bcl-2 cells (Supplementary Figure 2A). Conversely, 10 μg/ml BetA could trigger LC3 conversion, indicating that lower doses of BetA do not induce autophagy in the indicated cells. Under the same conditions, ATO and Dox alone could partially initiate LC3-II expression in IM-9 but not in IM-9/Bcl-2 cells. These results revealed that neither ATO nor Dox induce autophagy in IM-9/Bcl-2 cells. However, BetA may initiate autophagy in IM-9/Bcl-2 cells. BetA also induced LC3-II expression in U-266-1970 but not in 8226 cells (Supplementary Figure 2B).

The cellular localization of LC3 can also be evaluated by following cells transiently transfected with the fluorescent autophagy marker GFP-LC3. As illustrated in Figure 2B, BetA caused an accumulation of a punctuate fluorescent pattern in IM-9/Bcl-2 cells but not in IM-9 cells, indicating the redistribution of LC3 to autophagic structures; however, untreated cells presented diffuse staining, confirming once again that BetA activates the autophagic process in IM-9/Bcl-2 cells.

To further determine whether BetA induces autophagic cell death in cells with high levels of Bcl-2 expression, we treated cell lines with 3-MA, which is the most commonly used pharmacological inhibitor of autophagy, to block the formation of autophagosomes [40]. 3-MA treatment inhibited BetA-induced autophagosome formation in IM-9/Bcl-2 cells as measured by LC3 conversion and p62 degradation (Figure 2C). As a positive control, we used EBSS (starvation medium), a classical stimulus that induces the accumulation of autophagosomes and autophagic flux. Moreover, 3-MA treatment efficiently inhibited BetA-induced cell death in IM-9/Bcl-2 and U-266-1970 cells but not in IM-9 and 8226 cells (Supplementary Figure 2C).

To further confirm the effects of BetA, we knocked down ATG5 in cells, which is an important factor for autophagy [3] (Supplementary Figure 2D). ATG5 depletion obviously decreased LC3-II expression in BetA-treated IM-9/Bcl-2 and U-266-1970 cells (Supplementary Figure 2E). Furthermore, ATG5 deficiency obviously inhibited BetA-induced cell death in IM-9/Bcl-2 and U-266-1970 cells but not in IM-9 and 8226 cells (Supplementary Figure 2F). We then analyzed the rate of degradation of long-lived proteins in IM-9/Bcl-2 cells treated with BetA. As illustrated in Figure 2D, BetA treatment increased the degradation of long-lived proteins in IM-9/Bcl-2 cells cultured in complete medium. The lack of ATG5 expression restrained the degradation of these proteins. Under the same conditions, the degradation rate of long-lived proteins exhibited little change in treated IM-9 cells. These results suggest that BetA induces autophagic cell death in cells with high levels of Bcl-2 expression.

Increased detection of autophagic markers such as the conversion of LC3-I to LC3-II can result from either increased autophagy or inhibition of autophagic flux [41]. Bafilomycin A1 (BafA1), a drug that blocks the fusion of autophagosomes with lysosomes, can inhibit autophagic flux [42]. As Figure 2E illustrates, BafA1 treatment of BetA-treated IM-9/Bcl-2 cells led to the stabilization of p62. Furthermore, BetA-induced increases in the LC3-II levels were further enhanced upon inhibition of lysosomal activity (Figure 2E).

To better assess autophagic flux, the autophagy substrates LC3 and p62 were monitored in the presence of chloroquine (CQ), which blocks lysosome acidification, the degradation of autophagosome contents, and flux [43]. BetA-treated U-266-1970 cells accumulated LC3-II and p62 in the presence of CQ, which is indicative of high autophagic flux (Supplementary Figure 2B). These results suggest that either BetA or CQ is stimulating rather than inhibiting autophagy. Moreover, BafA1 and CQ treatment greatly ameliorated BetA-induced cell death in cells with high levels of Bcl-2 expression (Supplementary Figure 2G and 2H).

We then stained untreated and treated cells with monodansylcadaverine (MDC). The formation of autophagic vacuoles was assessed by detecting the increase of MDC fluorescence [44–46]. The MDC fluorescent intensity of BetA-treated IM-9/Bcl-2 cells was significantly increased compared with that in the untreated cells. However, the fluorescence in IM-9 cells showed no obvious changes (Figure 2F). Transmission electron microscopy experiments were also used to confirm the presence of classical autophagic structures in IM-9/Bcl-2 cells. BetA-treated cells had more typical autophagic structures than untreated cells (Figure 2G). Together, these results confirmed that BetA initiated non-apoptotic and autophagic cell death in cells with high levels of Bcl-2 expression.

### Beclin-1 is required for the BetA-induced stimulation of autophagic cell death n cells with high levels of Bcl-2 expression

**Figure 3:**
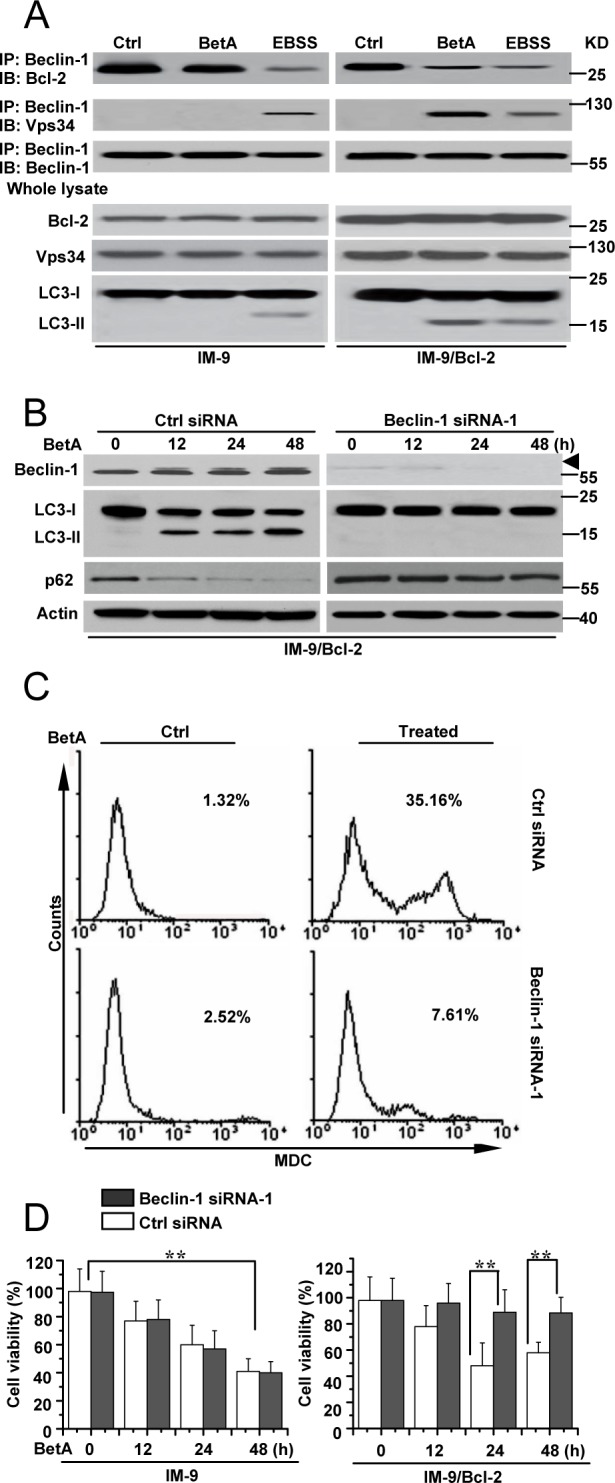
Beclin-1 is required for autophagic cell death in IM-9/Bcl-2 cells **(A)** Detection of Beclin-1/Bcl-2 complex and Beclin-1/Vps 34 complex association in IM-9/Bcl-2 cells, respectively. *Top row*, Cells were cultured for 24 h, either in complete medium (control) or in medium supplemented with 10 μg/ml BetA. EBSS treatment (12 h) was used as an autophagy control. Cells were lysed and were subjected to immunoprecipitation with anti-Beclin 1 antibody and protein G-Sepharose. The immunoprecipitates were subjected to immunoblotting using anti-Bcl-2, Vps34 or anti-Beclin 1 antibodies. *Bottom*, lysates were immunoblotted with the antibodies indicated. The Western blots are representative of three independent experiments. **(B)** IM-9/Bcl-2 cells were transfected with Beclin-1 or Ctrl siRNA for 48 h, and then treated with BetA for different periods of time. Immunoblot analysis of LC3-I and LC3-II levels, p62 and Beclin-1 expression. Arrowhead referred to the upshifted Beclin-1. β-Actin immunoblotting was used as a loading control. **(C)** IM-9/Bcl-2 cells were treated as described in **B**, and then treated cells were collected for MDC staining by flow cytometry. Representative results of three experiments with consistent results are shown. **(D)** IM-9 and IM-9/Bcl-2 cells were transfected with Beclin-1 or Ctrl siRNA for 48 h, and then treated with BetA for different periods of time. Cell viability was determined as described in Materials and methods. Graphs showing results of quantitative analyses (*n*=3, mean ± S.D. **,*P*<0.01, the compared groups: 48 h & 0 h under the same RNAi treatment in IM-9 cells; Beclin-1 siRNA-1 & Ctrl siRNA treatment at 24h or 48 h in IM-9/Bcl-2 cells). Representative results of three experiments with consistent results are shown.

Autophagy is tightly regulated by the activity of the Beclin-1 complex regarding the initiation of autophagosome formation [47]. Moreover, previous studies have shown that the anti-apoptotic protein Bcl-2 down-regulates autophagy by binding to Beclin-1. Dissociation of the Beclin-1/Bcl-2 complex stimulates autophagy [48]. Moreover, Beclin-1 interacts with the Vps34 class III phosphatidylinositol 3-kinase (Vps34) to form the Vps34 complex, which promotes autophagic flux [49, 50]. Therefore, we detected the dissociation of Beclin-1 and Bcl-2 as well as the interaction of Beclin-1 with Vps34 using co-immunoprecipitation experiments. Our data revealed that similar to starvation conditions, BetA induced the conversion of LC-I to LC3-II and the dissociation of the Beclin-1/Bcl-2 complex in IM-9/Bcl-2 cells but not in IM-9 cells [48] (Figure 3A). Meanwhile, the interaction of Beclin-1 with Vps34 was observed in IM-9/Bcl-2 cells but not in IM-9 cells. These results suggest that Beclin-1 may be important for autophagy.

Next, we compared the changes in the abundance of LC3-II and p62 caused by BetA treatment in IM-9/Bcl-2 cells following siRNA-mediated silencing of Beclin-1. Beclin-1 siRNA efficiently decreased Beclin-1 expression in the indicated cells (Supplementary Figure 3A). As expected, the increase in the abundance of LC3-II and the decrease of p62 caused by BetA was reduced by down-regulation of Beclin-1 expression in IM-9/Bcl-2 cells (Figure 3B). Similar results were observed in U-266-1970 cells (Supplementary Figure 3B). We next compared the MDC fluorescence intensity in BetA-treated IM-9/Bcl-2 cells following siRNA-mediated silencing of Beclin-1. Accordingly, our experiments revealed a marked decrease in the MDC fluorescence intensity when Beclin-1 expression was depleted (Figure 3C). Cell viability assays also demonstrated that depleting Beclin-1 expression obviously decreased cell death in treated cells with high levels of Bcl-2 expression (Figure 3D and Supplementary Figure 3C), indicating the requirement of Beclin-1 for stimulating autophagic cell death by BetA in cells with high Bcl-2 expression.

### DAPK mediates Beclin-1 phosphorylation to promote autophagic cell death

**Figure 4:**
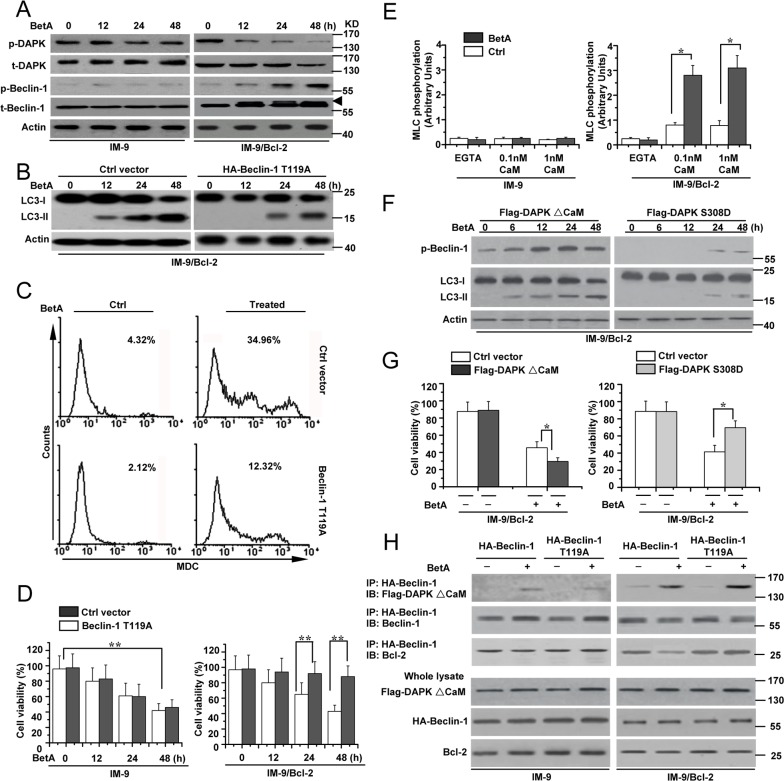
DAPK-mediated Beclin-1 phosphorylation contributes to BetA-induced autophagic cell death **(A)** IM-9 and IM-9/ Bcl-2 cells were treated with BetA for different periods of time. Treated cells were lysed for detecting DAPK phosphorylation (p-DAPK), total DAPK (t-DAPK), Beclin-1 phosphorylation (p-Beclin-1) and total Beclin-1 (t-Beclin-1) by Western blotting, with β-Actin serving as a loading control. Arrowhead referred to the upshifted Beclin-1. **(B)** IM-9/ Bcl-2 cells were transfected with Ctrl vector or HA-Beclin-1 T119A for 48 h, and then cells were lysed for detecting LC3-I and LC3-II levels by Western blotting, with β-Actin serving as a loading control. **(C)** IM-9/ Bcl-2 cells were treated with BetA for 48 h, and then treated cells were collected for MDC staining by flow cytometry. Representative results of three experiments with consistent results are shown. **(D)** IM-9/ Bcl-2 cells were treated as described in **B**, and cell viability was determined as described in Materials and methods. Graphs showing results of quantitative analyses (*n*=3, mean ± S.D. **,*P*<0.01, the compared groups: 48 h & 0 h under the same vector treatment in IM-9 cells; Beclin-1T119A & Ctrl vector treatment at 24 h or 48 h in IM-9/Bcl-2 cells). **(E)** Detection of DAPK activity. DAPK was immunoprecipitated with anti-DAPK antibodies from BetA-treated cells and an *in vitro* kinase assay was performed using MLC (2 mg) as a substrate in the presence of EGTA, 0.1 or 1 nM calmodulin (CaM). Kinase activity, calculated by quantifying the relative MLC phosphorylation levels after normalization to DAPK protein levels (*n*=3, mean ± S.D. *,*P*<0.05). **(F)** IM-9/Bcl-2 cells were transfected with Flag-DAPK S308D or Flag-DAPK ΔCaM for 48 h, and then cells were lysed for detecting p-Beclin-1, LC3-I and LC3-II levels by Western blotting, with β-Actin serving as a loading control. **(G)** IM-9/Bcl-2 cells were transfected with Flag-DAPK S308D or Flag-DAPK ΔCaM for 48 h, and then treated with BetA for 48 h. Cell viability was determined as described in Materials and methods. Graphs showing results of quantitative analyses (*n*=3, mean ± S.D. *,*P*<0.05). **(H)** Indicated cells were transfected with HA-tagged Beclin-1 (WT) or HA-tagged T119A Beclin-1 mutant with or without Flag-tagged activated DAPK (ΔCaM). Beclin-1 was immunoprecipitated using HA antibodies, and the co-immunoprecipitated proteins, as well as the total cell extracts, were blotted using the indicated antibodies. All data are representative of three independent experiments.

We then determined how Beclin-1 regulates autophagic cell death. We first examined Beclin-1 expression and found that Beclin-1 expression was similar in BetA-treated IM-9 and IM-9/Bcl-2 cells (Figure 4A and Supplementary Figure 4A). We then speculated that post-transcriptional modifications of Beclin-1 mediate autophagy. A previous study demonstrated that Beclin-1 phosphorylation regulated autophagy [51]. An upshifted band of Beclin-1 was observed in an immunoblotting assay in BetA-treated IM-9/Bcl-2 cells, which could represent phosphorylated Beclin-1 in treated IM-9/Bcl-2 cells (Figure 4A and Supplementary Figure 4A).

A previous study revealed that activated DAPK phosphorylates Beclin-1 at Thr 119 to regulate autophagy [51]. We then examined whether DAPK mediates Beclin-1 phosphorylation in cells with high levels of Bcl-2 expression. DAPK can be activated via dephosphorylation at Ser 308 [52, 53]. We used a specific antibody to detect the phosphorylation levels of DAPK (p-DAPK) at Ser308. We found that the levels of p-DAPK were decreased in treated IM-9/Bcl-2 and U-266-1970 cells but exhibited little changes in treated IM-9 and 8226 cells. Meanwhile, the expression of total DAPK (t-DAPK) showed no obvious change among all the cell lines (Figure 4A and Supplementary Figure 4A). We also used an antibody to detect the levels of phosphorylated Beclin-1 (p-Beclin-1) at Thr119. p-Beclin-1 increased over time in treated cells, and the levels were inversely associated with the changes in the p-DAPK levels (Figure 4A and Supplementary Figure 4A).

To confirm that Beclin-1 phosphorylation is required for autophagic cell death, we constructed an HA-Beclin-1 T119A mutant in which Thr 119 was replaced with alanine (Supplementary Figure 4B). A previous study revealed that the mutant T119A BH3 peptide of Beclin-1 can inhibit its phosphorylation levels. Meanwhile, expression of the mutant decreased Beclin-1-mediated autophagy [51]. Our experiments revealed that the Beclin-1 T119A mutant decreased the conversion of LC3-I to LC3-II (Figure 4B) and the MDC fluorescence intensity in treated IM-9/Bcl-2 cells (Figure 4C). The cell viability assay also revealed that the mutant decreased autophagic cell death in treated IM-9/Bcl-2 cells (Figure 4D). IM-9 cells were used as a control. These results demonstrated that phosphorylation of Beclin-1 at Thr119 is necessary for autophagic cell death.

We further examined whether DAPK mediates Beclin-1 activation by regulating the phosphorylation status of Beclin-1. Our data showed that DAPK was dephosphorylated based on the changes of the phosphorylation status at Ser 308 (Figure 4A), suggesting that DAPK is activated. This was supported by measuring kinase activity *in vitro* using myosin II regulatory light chain (MLC) as a substrate. In these assays, endogenous DAPK protein was immunoprecipitated with antibodies targeting the C-terminus of the protein, which recognize both phosphorylated and nonphosphorylated DAPK [52]. DAPK immunoprecipitated from treated IM-9/Bcl-2 cells exhibited significantly higher kinase activity in the presence of 0.1–1 nM calmodulin than DAPK extracted from either IM-9 or untreated IM-9/Bcl-2 cells (Figure 4E).

We then constructed FLAG-DAPKΔCaM (an activated form of DAPK lacking its CaM-regulatory domain) [51, 54] and FLAG-DAPK S308D mutants (an inactive form of DAPK due to the mutation of Ser308 to Asp) [54, 55] (Supplementary Figure 4C). We first transfected either FLAG-DAPKΔCaM or FLAG-DAPK S308D into the cell lines. In BetA-treated cells, FLAG-DAPK S308D expression obviously decreased the levels of phosphorylated Beclin-1 and the conversion of LC3-I to LC3-II compared to FLAG-DAPKΔCaM expression (Figure 4F and Supplementary Figure 4D). Meanwhile, a marked decrease in the MDC fluorescence intensity was observed in cells transfected with FLAG-DAPK S308D compared to cells transfected with FLAG-DAPKΔCaM (Supplementary Figure 4E). Moreover, FLAG-DAPKΔCaM obviously enhanced cell death with BetA treatment, while FLAG-DAPK S308D restrained cell death (Figure 4G).

We then co-transfected FLAG-DAPKΔCaM with either HA-Beclin-1 or HA-Beclin-1 T119A into the available cells. Co-immunoprecipitation assays revealed that DAPKΔCaM associated with HA-Beclin-1 and HA-Beclin-1 T119A. However, DAPKΔCaM reduced the amount of Bcl-2 immunoprecipitated in treated IM-9/Bcl-2 cells expressing HA Beclin-1. Under the same conditions, DAPKΔCaM had no effect on the levels of Bcl-2, which co-immunoprecipitated with the T119A Beclin-1 mutant (Figure 4H). IM-9 cells were used as a control. Thus, a mutation at Thr 119 results in a stronger interaction between Beclin-1 and Bcl-2, which then becomes resistant to DAPK-dissociating effects, indicating that DAPK regulates Beclin-1 activation in autophagy via phosphorylation of Thr 119. Moreover, the Beclin-1 Thr 119 mutant also exhibited a reduction in BetA-induced cell death in cells expressing DAPKΔCaM (Supplementary Figure 4F). These results confirmed that DAPK mediates Beclin-1 phosphorylation to promote autophagic cell death in cells with high levels of Bcl-2 expression.

### Inactivation of Akt is required in BetA-induced apoptosis in cells with low levels of Bcl-2 expression

**Figure 5:**
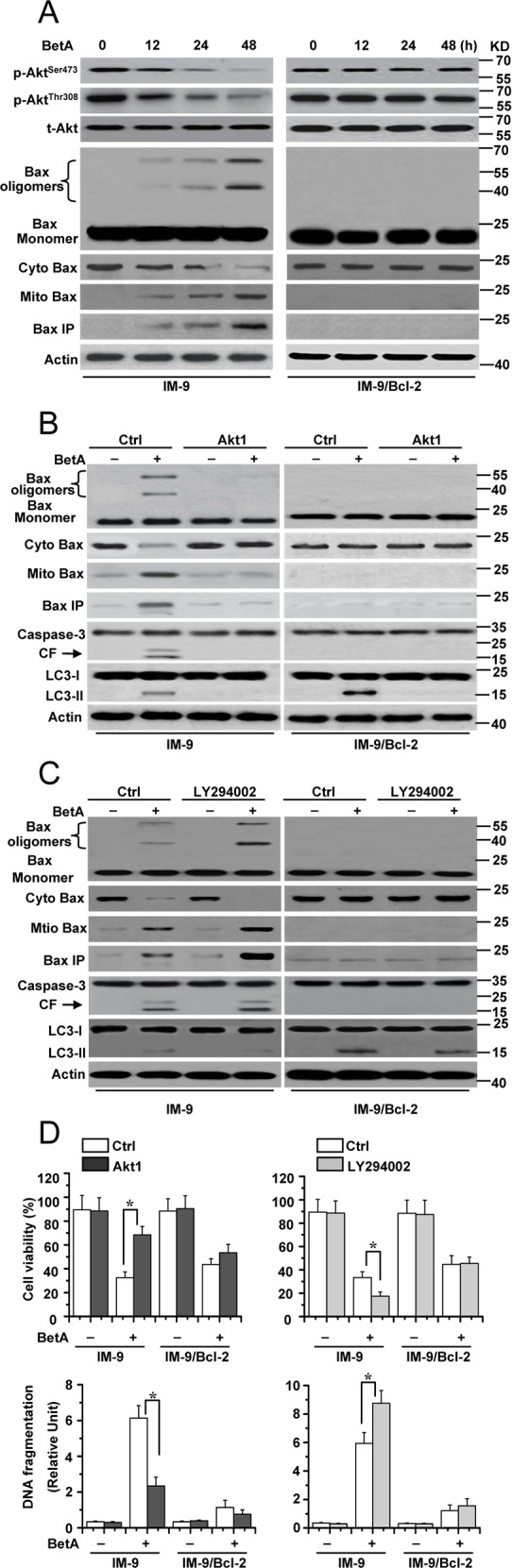
Akt inactivation mediates apoptosis in IM-9 cells **(A)** IM-9 and IM-9/ Bcl-2 cells were treated with BetA for different periods of time. Treated cells were lysed for detecting Akt phosphorylation (p-Akt), total Akt (t-Akt), Bax oligomerization, conformational change and mitochondrial translocation by Western blotting, with β-Actin serving as a loading control. Bax conformational change was detected as described before (Hu et al., 2012b). **(B)** IM-9 and IM-9/ Bcl-2 cells were transfected with the constitutively active Akt1 for 48 h, and then treated with BetA for 48 h. treated cells were lysed for Western blotting. **(C)** Cells were treated with BetA and/or 25 mM LY294002 for 72 h, and then cells were lysed and assayed for individual protein levels by Western blot. **(D)** Cells were treated with BetA for 48 h. *Top row*, treated cells were collected for detection of cell viability. Graphs showing results of quantitative analyses (*n*=3, mean ± S.D. *,*P*<0.05). *Down*, Apoptotic cell death was quantitatively detected by a cell death ELISA kit as described in Materials and methods. Graphs showing results of quantitative analyses (*n*=3, mean ± S.D. *,*P*<0.05). Representative results of three experiments with consistent results are shown.

We then examined the mechanisms of BetA-induced apoptosis in cells with low levels of Bcl-2 expression. In BetA-treated IM-9 cells, we observed changes in the Bax conformation, mitochondrial translocation and oligomerization accompanied by Akt dephosphorylation (Figure 5A). Meanwhile, BetA also induced casapse-3 cleavage and changes in Akt phosphorylation in 8226 cells (Supplementary Figure 5A). Considering that Akt is an upstream anti-apoptotic regulatory molecule [9, 56–58], we speculated that inactivation of Akt is required for BetA-induced apoptosis.

To confirm our speculation, we transfected a constitutively active Akt1 construct into the indicated cells to further examine the effect of Akt on apoptosis (Supplementary Figure 5B). We found that Akt overexpression obviously inhibited Bax oligomerization, conformational changes, mitochondrial translocation and caspase-3 cleavage in IM-9 cells (Figure 5B). Unsurprisingly, Akt1 overexpression prevented LC3 conversion in IM-9/Bcl-2 cells because Akt induction can restrain autophagy [59].

To further confirm the effects of Akt in apoptosis, we treated cells with LY294002, a PI-3K inhibitor, to inhibit the phosphorylation of Akt via upstream signaling molecules and examined the pro-apoptotic effect of Akt in cancer cells. We found that LY294002 efficiently enhanced Bax activation in cells after BetA treatment (Figure 5C). Moreover, Akt1 overexpression in BetA-treated IM-9 cells obviously increased cell viability and decreased apoptosis. LY294002 treatment showed opposing results (Figure 5D). Together, these results demonstrated that Akt mediates BetA-induced apoptosis in cells with low levels of Bcl-2 expression.

### PP2A is a key molecule that mediates BetA-induced apoptosis and autophagic cell death

**Figure 6:**
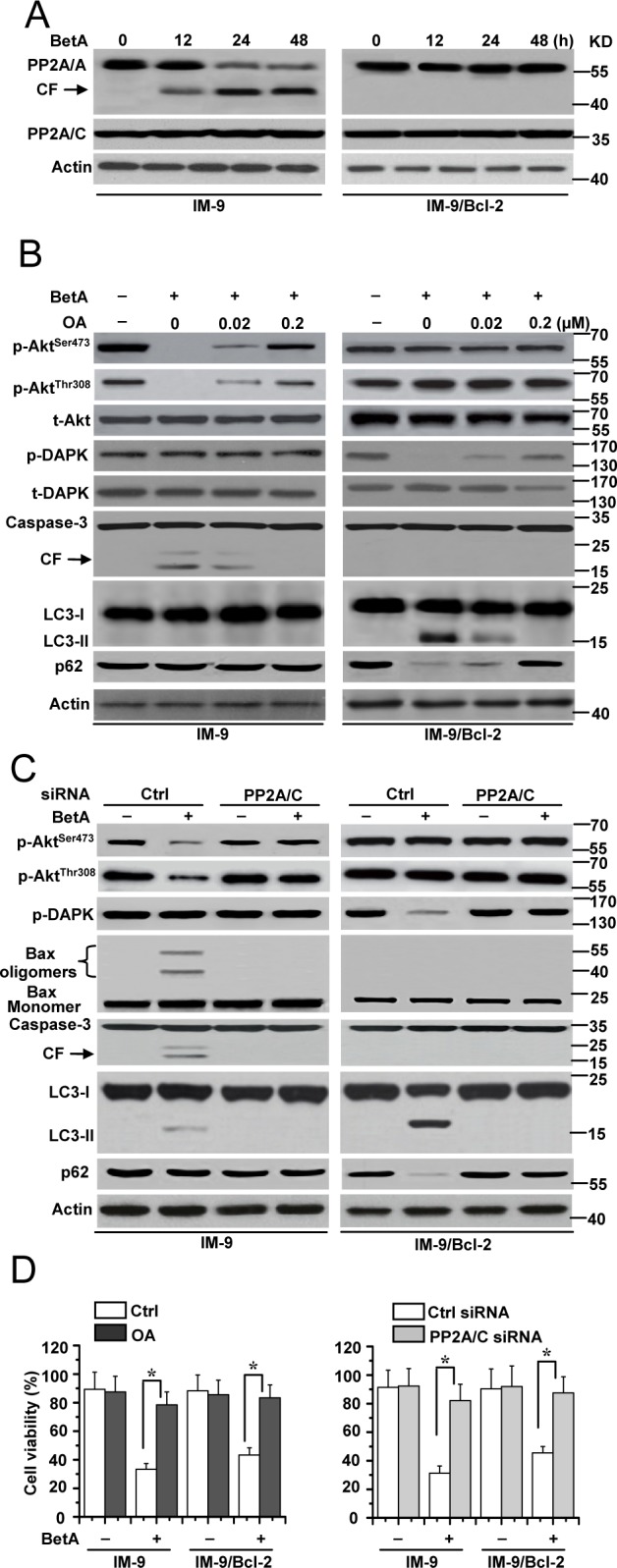
PP2A is required for BetA-induced apoptosis or autophagic cell death **(A)** Cells were treated by BetA for different periods of time, and then lysed for Western blotting with β-Actin serving as a loading control. Treated cells were lysed with sample buffer and subjected to immunoblot assay with an A subunit-specific anti-PP2A/A antibody. CF is referred to cleaved PP2A/A. The membrane was then stripped and reprobed with a C subunit-specific anti-PP2A/C antibody. β-Actin was used as a loading control. **(B)** Different cells were pre-incubated with indicated concentrations of OA for 1 h and exposed to BetA for 48 h, then lysed for detection. **(C)** Cells were transiently transfected with PP2A/C or Ctrl siRNA for 48 h, and then treated by BetA for Western blot analysis. **(D)** Cells were treated with OA or transfected with PP2A/C siRNA, and then treated with BetA for 48 h. Treated cells were collected for detection of cell viability. Graphs showing results of quantitative analyses (*n*=3, mean ± S.D. *,*P*<0.05). All data are representative of three independent experiments.

We found that the A subunit of PP2A (PP2A/A) was cleaved in cells with low levels of Bcl-2 expression but not in cells with high levels of Bcl-2 expression during the time course tested. The expression of the C subunit of PP2A (PP2A/C) showed little changes after treatment (Figure 6A and Supplementary Figure 6A). Importantly, we found that PP2A activity was increased in both IM-9 and IM-9/Bcl-2 cells (Supplementary Figure 6B). Along with other studies, we demonstrated that PP2A down-regulates Akt kinase activity via caspase-3-dependent pathways to induce apoptosis [60, 61]. Meanwhile, PP2A can mediate DAPK activation in cell death [53, 62]. Therefore, these results prompted us to speculate that PP2A is an important switch factor for regulating apoptosis and autophagic cell death in humans.

We first pre-treated cells with okadaic acid (OA), an inhibitor of PP2A. OA treatment in IM-9 cells resulted in a gradual reversal of BetA-induced Akt dephosphorylation and caspase activity in a dose-dependent manner. At the same time, OA rescued DAPK phosphorylation and p62 expression as well as decreased the conversion of LC3 in BetA-treated IM-9/Bcl-2 cells. However, OA treatment could not change the phosphorylation status of DAPK in IM-9 cells and of Akt in IM-9/Bcl-2 cells (Figure 6B).

To further determine the role of PP2A in our study, we knocked down the C subunit of PP2A (PP2A/C) (Supplementary Figure 6C) to interfere with PP2A function as previously described [63]. As depicted in Figure 6C and Supplementary Figure 6D, inhibiting PP2A/C expression restrained Bax oligomerization, caspase-3 activity and the dephosphorylation of Akt in IM-9 and 8226 cells, whereas PP2A/C depletion had little effect on the levels of phosphorylated DAPK and LC3 conversion in BetA-treated cells with low levels of Bcl-2 expression. Meanwhile, PP2A/C inhibition reduced LC3 conversion, p62 degradation and the dephosphorylation of DAPK in IM-9/Bcl-2 and U-266-1970 cells. In contrast, the Akt phosphorylation status was not affected in treated cells with high levels of Bcl-2 expression. Moreover, either OA or PP2A treatment obviously decreased cell death in both IM-9 and IM-9/Bcl-2 cells (Figure 6D). These results demonstrated that PP2A is the upstream regulatory molecule of apoptosis and autophagic cell death via regulation of Akt and DAPK phosphorylation, respectively.

**Figure 7:**
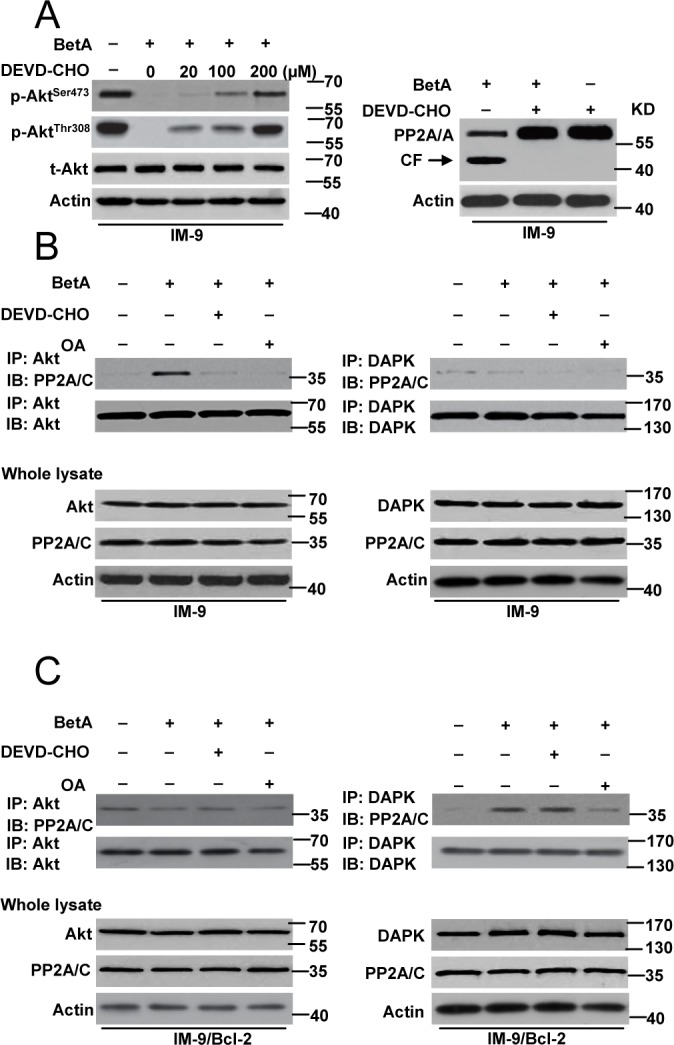
PP2A mediates autophagic cell death or apoptosis through regulating its connection with Akt and DAPK **(A)** *Left row*, Cells were pre-incubated with indicated concentrations of DEVD-CHO for 1 h and exposed to BetA for 48 h, then lysed in sample buffer for detection. Western blot analysis of t-Akt and p-Akt levels was shown. *Right*, Western blot analysis of the effect of DEVD-CHO (100 μM) in PP2A cleavage in IM-9 cells. **(B)** Detection of the binding of Akt or DAPK with PP2A in IM-9 cells. Cells were incubated with or without BetA for 48 h. In some groups of cells, 0.2 μM of OA or 100 μM of DEVD-CHO was added 1 h prior to the addition of drug. The cells were then lysed with for immunoprecipitation with anti-Akt or anti-DAPK antibody followed by immunoblot assay with anti- PP2A/C and anti-Akt or anti-DAPK antibodies. **(C)** Detection of the binding of Akt or DAPK with PP2A in IM-9/Bcl-2 cells. Cells were incubated with or without BetA for 48 h. In some groups of cells, 0.2 μM of OA or 100 μM of DEVD-CHO was added 1 h prior to the addition of drug. The cells were then lysed with for immunoprecipitation with anti-Akt or anti-DAPK antibody followed by immunoblot assay with anti- PP2A/C and anti-Akt or anti-DAPK antibodies. Representative results of three experiments with consistent results are shown.

We then examined the detailed mechanisms of PP2A mediated-apoptosis and autophagic cell death. We first determine whether caspase-3 mediates Akt dephosphorylation and the proteolysis of the A subunit associated with increased PP2A activity in IM-9 cells. We pre-treated cells with DEVD-CHO, a specific caspase-3 inhibitor. The results indicated that in IM-9 cells, inhibiting caspase-3 activation restored Akt phosphorylation in the presence of BetA and inhibited PP2A proteolysis (Figure 7A). Moreover, the PP2A-Akt association [60] was greatly improved after treatment with BetA, whereas this interaction was drastically decreased upon inhibition of caspase-3 and PP2A activation in IM-9 cells (Figure 7B). However, the PP2A-DAPK association [62] was not found in IM-9 cells.

In contrast, the PP2A-DAPK association was observed in IM-9/Bcl-2 cells. This interaction was obviously reduced in the presence of OA but not DEVD-CHO. As expected, Akt-PP2A was not found in IM-9/Bcl-2 cells (Figure 7C).

Together, our data revealed that in BetA-treated myeloma cells, PP2A could mediate different types of cell death by interacting with either Akt or DAPK.

### BetA induces apoptosis in IM-9 xenografts and autophagy in IM-9/Bcl-2 xenografts *in vivo*

**Figure 8:**
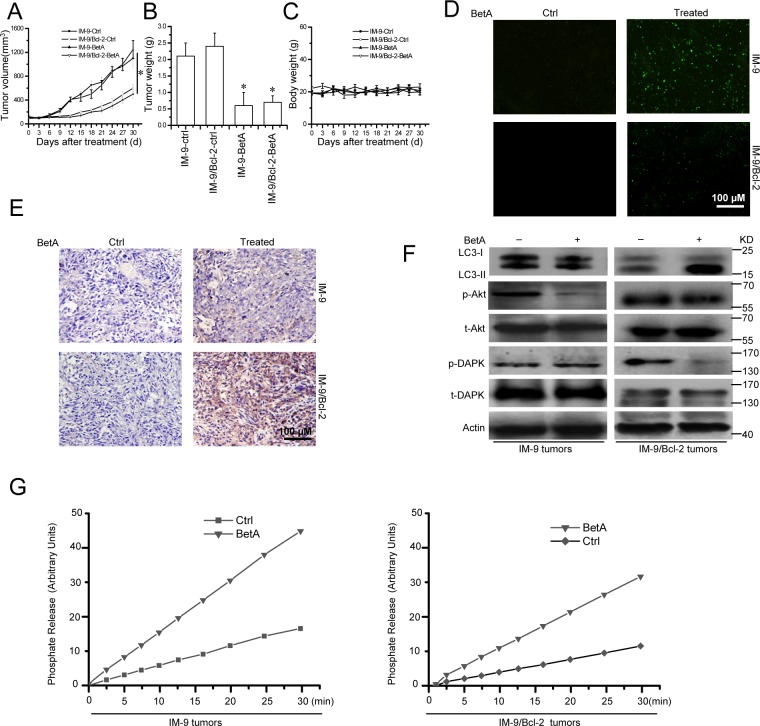
BetA inhibits tumor growth and induces apoptosis or autophagic cell death in IM-9 or IM-9/Bcl-2 xenograft *in vivo* **(A)** The cell lines were subcutaneously injected into the right dorsal flank of 6- to 8-week-old female athymic nude BALB/c mice. Following tumor growth for 7 days, the tumor-bearing mice were randomly assigned into the following two groups (10 mice per treatment group): Ctrl group and BetA-treated group. Mice were injected intraperitoneally with BetA as described in the Materials and Methods section. Tumor size was measured at the indicated days and volume was calculated (*,*P*<0.05). **(B)** After the experiments are stopped, the tumor weight change of animals was measured. In tumor model, significant differences in tumor weight in mice treated with BetA versus Ctrl are shown (*,*P*<0.05). **(C)** Body weights of IM-9 or IM-9/Bcl-2 mice models were plotted at 3-day intervals. There were no significant differences in weights among the different groups (P>0.05). Values were shown as mean±S.D. **(D)** TUNEL assay in tumor models. Dissected tumors were weighed and fixed in 4% paraformaldehyde in PBS, embedded in paraffin and cut into 3–5 μm sections. The sections were used for TUNEL experiments. Tumor tissue preparation and procedure for TUNEL staining is described in the Materials and Methods section. Representative micrograph of TUNEL assay was shown. Bars, 100 μM. **(E)** LC3-II immunohistochemical staining in tumor model. As described in **D**, the paraffin-embedded tumor samples were subjected to immunohistochemical staining with a polyclonal anti-LC3 antibody. Representative micrograph of immunohistochemical staining with anti-LC3 antibody in BetA -treated and control tumors was shown. Bars, 100 μM. **(F)** Effect of BetA on the expression of LC3-II, total Akt (t-Akt), Akt phosphorylation (p-Akt), total DAPK (t-Akt) and DAPK phosphorylation (p-DAPK). After treatment with 12 mg/kg BetA as described in the Materials and Methods section, tumor samples were harvested, and proteins were prepared and subjected to immunoblot analysis using the corresponding antibodies. β-Actin was used as a loading control. **(G)** Tumor lysates were used to measure PP2A activity as described in the Materials and Methods section. All data are representative of three independent experiments.

To investigate the anti-tumor efficacy of BetA *in vivo*, we used IM-9 and IM-9/Bcl-2 as model tumor cells. We injected IM-9 and IM-9/Bcl-2 cells into nude mice to establish xenografted tumors. Tumor-bearing mice were treated with BetA, and the tumor volumes were measured every 3 days for 30 days. We found that after 30 days, the IM-9 and IM-9/Bcl-2 tumors treated with BetA exhibited slower growth and were generally half the size of the untreated tumors (Figure 8A). Similar results were also found regarding the tumor weight (Figure 8B). To evaluate the possible adverse effects of BetA, the weight of the mice was monitored every 3 days throughout the entire experiment. The weight curve of the BetA-treated group was highly similar to that of the control group (Figure 8C). Neither ruffled fur nor toxic death was observed in the BetA-treated group.

To investigate the mechanism of BetA-induced tumor growth inhibition *in vivo*, mice were sacrificed after the final BetA dose, and tumor tissues were collected. Tumor cell apoptosis was assessed by terminal deoxynucleotidyltransferase-mediated dUTP nick-end labeling (TUNEL) staining; autophagy was assessed by LC3 expression using immunohistochemical staining and immunoblotting analysis. As shown in Figure 8D, a significantly greater percentage of TUNEL-positive nuclei were observed in IM-9 tumors treated with BetA than in tumors from the Ctrl group. In contrast, there were few differences in the percentage of TUNEL-positive cells between the BetA-treated IM-9/Bcl-2 tumors and the corresponding control tumors (Figure 8D).

At the same time, the expression of total cytoplasmic LC3-II detected by immunohistochemical staining was markedly elevated in BetA-treated IM-9/Bcl-2 tumors than in control tumors (Figure 8E). Conversely, little LC3-II staining was observed in BetA-treated IM-9 tumors. Consistent with this, the immunoblotting analysis revealed that the LC3-II levels were higher in BetA-treated IM-9/Bcl-2 tumors than in IM-9 tumors (Figure 8F). Meanwhile, opposite changes of Akt and DAPK phosphorylation were observed in treated IM-9 and IM-9/Bcl-2 tumors, respectively (Figure 8F). Moreover, we found that PP2A activity was increased in IM-9 and IM-9/Bcl-2 tumors (Figure 8G). These data suggest that both apoptosis and autophagy are associated with BetA-induced tumor growth inhibition *in vivo*.

## DISCUSSION

The results described in this study provide evidence that autophagy can be an adaptive mechanism that contributes to tumor cell death. Bcl-2 overexpression results in the inhibition of tumor cell apoptosis. However, myeloma cells still undergo cell death and display characteristics of active autophagy. Impaired autophagy with the autophagosome inhibitor 3-MA perhaps correlated with enhanced cell survival. This appears to be due to a direct anti-survival effect of autophagy in tumor cells since autophagy inhibition by Beclin-1 siRNA enhances tumor cell growth and suppresses tumor cell death *in vitro*.

BetA is a cytotoxic plant-derived compound that is tumor-specific and does not kill normal cells [64–66]. A previous study revealed that BetA is a promising anti-cancer agent with apoptosis-inducing effects that acts on the permeability transition pore (PT-pore) in a Bax/Bak-independent manner [33]. Recently, we found that BetA could initiate Puma-dependent apoptosis in chemoresistant ovarian cancer cells [66]. Here, we show for the first time that BetA can induce autophagic cell death in myeloma. Autophagic flux was found to be functional and led to enhanced degradation of long-lived proteins, the conversion of LC3 and the degradation of p62 proteins. Our observations contrast to previous findings by Yang *et al*. [67], who reported that BetA induces apoptosis and blocks ongoing autophagy (i.e., decreases autophagic flux) in human multiple myeloma cells as measured by the accumulation of p62 protein. We found that p62 levels were increased at an earlier time point but rapidly degraded at later time points, suggesting that BetA causes the induction of autophagic flux. Treatment with BafA13, MA or CQ further demonstrated that BetA could enhance autophagic flux. Moreover, our report reveals that BetA can initiate either apoptosis or autophagic cell death in the same types of myeloma cells.

The data from this study and from Potze *et al*. [34] reveal that BetA can initiate autophagic flux in tumor cells. Potze's group has also demonstrated that blocking apoptosis by inhibiting caspase does not inhibit BetA-induced cell death. They speculated there exists a caspase-independent cell death pathway, and their report indicates that autophagy serves as a rescue pathway instead of an alternative cell death pathway in BetA-treated cells. However, our experiments confirm that BetA indeed triggers caspase-independent cell death, which is autophagic cell death. Similar to BetA, B10, a derivative of BetA, induced cell death in an apoptosis-dependent and apoptosis-independent manner [42]. Our findings slightly differ from the results observed with B10 treatment. Gonzalez's report indicated that B10 induces autophagy and abrogates autophagic flux. In contrast, our report confirms that BetA initiates autophagic flux towards cell death. Of course, we should study the more basic mechanisms of BetA-mediated apoptosis and autophagy. It is possible that BetA-mediated autophagy may result in enhanced lipid modification, which initiates cell death [68]. We revealed that BetA can induce different types of cell death in myeloma cells. Tumor experiments *in vivo* also demonstrated this conclusion. Moreover, we elucidated the detailed mechanism of BetA-induced cell death, indicating that PP2A is as pivotal mediator in regulating apoptosis and autophagic cell death in multiple myeloma cells.

Previous studies have highlighted the regulatory function of PP2A in apoptosis and autophagy [53, 60, 61]. We showed for the first time how PP2A regulates apoptosis and autophagic cell death in the same types of tumor models. Gozuacik *et al*. found that the PP2A-DAPK pathway mediates caspase-3 activation and an autophagic cell death process that acts in concert with apoptosis [53]. However, our experiments revealed that BetA could induce caspase-3-dependent or caspase-3-independent PP2A activity. Activated caspase-3 could cleave PP2A/A to enhance PP2A activation [27, 60, 61] in cells with low levels of Bcl-2 expression. Cleaved PP2A binds with Akt to promote apoptosis [60, 61]. Bcl-2 overexpression inhibits caspase-3 activity, and caspase-3 inactivation mitigates the degradation of PP2A/A, resulting in the dissociation of PP2A and Akt. When isolated from Akt, PP2A binds to DAPK in caspase-3-independent manner to initiate autophagic cell death in cells with high levels of Bcl-2 expression. Therefore, although the B subunit of PP2A/B (PP2A/B) associates with DAPK [62], the integrity of PP2A/A may contribute to the association of PP2A with DAPK.

In summary, we report that BetA-induced cell death can induce either apoptosis or autophagic cell death processes under different conditions. Moreover, we illuminated the molecular mechanism of PP2A as a regulatory factor to mediate apoptosis and autophagic cell death. These novel properties of BetA are expected to have important implications for implementing BetA-based therapeutics, particularly against apoptosis-resistant cancers.

## MATERIALS AND METHODS

### Materials

Arsenic trioxide (ATO) (202673), BetA (betulinic acid) (B8936), Doxorubicin (Dox) (D1515), 3-methyladenine (3-MA) (M9281), okadaic acid (OA) (O1506), Bafilomycin A1 (BafA1) (B1793), DEVD-CHO (A0835), LEHD-CHO (SCP0095), IETD-CHO (A1216) and Annexin V (A9210) were supplied by Sigma (St. Louis, MO, USA). LC3B (L7543), DAPK (D2178), phosphor-DAPK (Ser308) (D4941), FLAG (F7425) and actin (clone AC-74, A5316) antibodies were obtained from Sigma. Alkaline phosphatase (AP) (M0290S) was purchased from New England Biolabs (Beverly, MA, USA). Bismaleimidohexane (BMH) (22330) was obtaine from Pierce (Rockford, IL, USA). phospho-Akt (Ser 473) (clone 587F11, #4051), phospho-Akt (Thr308) (#13038), p62 (#88588), Akt (#9272), PP2A/C (52F8)(#2259), PP2A/A (#2039), ATG5 (#12994), caspase-3 (clone 8G10, #9665) and Cox IV (#4844) were purchased from Cell Signaling (Beverly, MA, USA). Cyt c (sc-13156), Beclin-1 (E-8) (sc-48341), Bcl-2 (sc-7382), HA (F-7) (sc-7392), and Bax N-20 (sc-493) antibodies were from Santa Cruz (Santa Cruz, CA, USA). Vps34 (AP8014a) and phospho-Beclin-1 (Thr119) (AP3765a) antibodies were purchased from Abgent (San Diego, CA, USA). Earle's balanced salt solution (EBSS) (14155-063) was from Gibco (Carlsbad, CA, USA).

### Gene silencing with small interfering RNAs and plasmids

The siRNA oligonucleotides (purchased from Dharmacon (Lafayette, CO, USA)) are: Beclin-1 siRNA-1, starting at positions 353 (5′-CUCAG GAGAGGAGCCAUUU-3′); Beclin-1 siRNA-2, starting at positions 1005 (5′-CAGUUUGGCACAAUCAAUA-3′) (NM_001313998.1); ATG5 siRNA-1, starting at positions 415 (5′-GUGAGAUAUGGUUUGAAUA-3′); ATG5 siRNA-2, starting at positions 671 (5′-GCAAC UCUGGAUGGGAUUG-3′) (NM_001286106.1). Human PP2A/C small interfering RNA (siRNA; sc-43509), which consists of a pool of three target-specific 19 to 25 nt siRNA and the nonsilencing control siRNA (sc-37007) was purchased from Santa Cruz Biotechnology, Inc. Beclin-1 and DAPK constructs were generated by RT–PCR from total RNA isolated from cells and cloning of the RT–PCR products into pHA (OGS3215) (Up primer: atagaattctATGGAAGGGTCTAAGACGTC; Down primer: ataggtaccTCATTTGTTATAAAATTGTGAGGAC) and pFLAG-CMV-2 expression vector (E7033) (Sigma) (Up primer: tatgaattcaATGACCGTGTTCAGGCAG; Down primer: aatggtaccTCACCGGGATACAACAGAG) using *EcoR*I and *Kpn*I, respectively. HA-Beclin-1 T119A, Flag-DAPK -CaM (an activated form of DAPK lacking its CaM-regulatory domain) and Flag-DAPK S308D (an inactive form of DAPK due to the mutant of Ser308 to Asp) mutants were generated by site-directed mutagenesis using *Pfu*-ultra polymerase (Stratagen, La Jolla, CA, USA) followed by *DpnI* digestion (Fermentas Inc., Glen Burnie, MD, USA) according to the manufacturer's instructions. The constitutively active Akt1 construct HA-PKB-T308D/S473D was obtained as previously described [56]. GFP-LC3 construct was a gift from Quan Chen (Chinese Academy of Sciences, Beijing, China).[69]

### Cell culture and transfection

IM-9, 8226, U-266-1970 cells were obtained from the American Type Culture Collection (ATCC, Manassas, VA, USA). IM-9/Bcl-2 cells [35] were the gift from Quan Chen (Chinese Academy of Sciences, Beijing, China). Cells were cultured with DMEM media (Sigma) supplemented with 10% fetal bovine serum (Hyclone, Logan, UT, USA) and 1% penicillin–streptomycin at 37°C under 5% CO_2_.

For transfection, cells were seeded on 6-well plates and then transfected with the appropriate siRNA or plasmids using the manufacturer's protocols. Typically, cells were seeded on coverslips in the 6-well plates, and then 100 nM siRNA and 4 μl of DMRIE-C reagent (Invitrogen, Carlsbad, CA, USA) were used per coverslip. The cells were incubated for 4 h in the transfection mixture, which was then replaced with fresh culture medium.

### Assay of cell viability and death

For assay of cell viability, cells were treated with BetA for the indicated time. After treatment, cell viability was detected by CellTiter-Glo Luminescent Cell Viability Assay from Promega as described before [70, 71]. Two methods were used to assess BetA-induced apoptotic cell death: detection of DNA fragmentation with the Cell Death Detection ELISA kit (Roche Diagnostics, Castle Hill, NSW, Australia) and Western blot analysis of caspase-3 cleavage as well as Cyt c release. The Cell Death Detection ELISA quantified the apoptotic cells by detecting the histone-associated DNA fragments (mono- and oligo-nucleosomes) generated by the apoptotic cells [56]. For Annexin V assay, cells were treated and then stained with Annexin V for flow cytometry detection (Becton Dickinson, Franklin Lakes, NJ).

Autophagic activity was examined by five methods: MDC staining; long-lived protein degradation; electron microscopy; GFP-LC3 assay; immunoblot analysis of the conversion of LC3-I to LC3-II. MDC was used to evaluate the abundance of autophagic vacuoles in cells as previously reported [72]. A 10 mM stock solution of MDC was prepared in Me_2_SO. Following treatment, cells were stained with MDC at a final concentration of 10 μM, for 10 min at 37°C. After washing with phosphate-buffered saline, cells were collected for analyzing the fluorescence intensity by flow cytometry. Protein degradation was measured as previously described [72]. Briefly, the cells were radiolabeled for 24 h with 0.05 mCi/ml of L-[U-^14^C] valine. At the end of the labeling period cells were rinsed three times with phosphate-buffered saline and then incubated for 6 h with 10 mM valine full medium either in the presence or in the absence of BetA or EBSS. Electron microscopy was examined as followings: the cells were fixed for 1 h at 4°C in 1.6% glutaraldehyde in 0.1 M Sorensen phosphate buffer (pH 7.3), washed, fixed again in aqueous 2% osmium tetroxide, then dehydrated in ethanol, and embedded in Epon. Ultrathin sections stained with uranyl acetate and lead citrate were then processed for electron microscopy with a Zeiss EM 902 transmission electron microscope at 80 kV. GFP- LC3 assay was performed in IM-9/Bcl-2 cells transiently transfected with GFP-LC3 constructs, and then cells were cultured on coverslips and then treated with or without morphine for the indicated times. Fluorescence was immediately observed using an Olympus DP72 microscope (Olympus Corporation, Tokyo, Japan).

### Cell fractionation, immunoprecipitation and western blot analysis

Mitochondria and cytoplasm from cells were fractionated by differential centrifugation as previously described [73]. For immunoprecipitation, all cells were harvested by resuspension in CHAPS cell extract buffer (Cell Signaling) and sonicated on ice. Lysates were centrifuged at 14,000 × g at 4°C for 15 min. Cell extracts were precleared and incubated with antibodies against Bax, Beclin-1, HA, Akt or DAPK with protein A-Sepharose (Invitrogen) to pull down immune complexes. The Sepharose was washed three times with lysis buffer and two times with PBS. Cytosol, mitochondria, total lysates, and immunoprecipitates were analyzed by western blot [74] with antibody dilutions as follows: actin at 1:20 000; p-Akt, Akt, p-DAPK, DAPK, Beclin-1, p-Beclin-1, caspase-3, endoG, HA, Flag, Akt, p-Akt, Vps34, CoxIV at 1:2000; and Bax, Bcl-2, LC3, Cyt, PP2A/A, PP2A/C c at 1:1000.

### *In vitro* kinase assays treatment

Endogenous DAPK was immunoprecipitated as described above using protein A-agarose beads bound to a rabbit polyclonal antibody raised against the DAPK C-terminal tail region (sc-10805, Santa Cruz). Beads were incubated for 30 min at 30°C in 30 μl reaction buffer (50 mM HEPES pH 7.5, 8 mM MgCl_2_, 2 mM MnCl_2_ and 0.1 mg/ml BSA, 0.5 mM CaCl_2_, 50 mM ATP, 2 mg MLC (Sigma-Aldrich))containing 15 mCi [γ-^32^P]ATP, and the indicated amounts of bovine calmodulin (Sigma-Aldrich) or 5mM EGTA. Samples were analyzed by immunoblotting and autoradiography. An *in vitro* kinase assay as described previously [53].

### Bax oligomerization

Bax oligomerization with cross-linking was detected as described previously [73]. Cells were washed with conjugating buffer containing 150 mM NaCl, 20 mM Hepes (pH 7.2), 1.5 mM MgCl2, and 10 mM glucose. Disuccinimidyl suberate in DMSO was added from a 10-fold stock solution to a final concentration of 2 mM. The samples were incubated at room temperature for 30 min with non-reducing buffer, and the cross-linker was then quenched by the addition of 1 M Tris-HCl (pH 7.5) to a final concentration of 20 mM and incubation at room temperature for 15 min. The samples were then solubilized in 0.5% Nonidet P-40 lysis buffer without a reducing agent and centrifuged at 12,000 × g for 10 min. Bax was detected by Western blot using a Bax polyclonal antibody.

### Antitumor activity *in vivo*

*In vivo* experiments were performed according to our previous report [9]. In brief, 2 × 10^6^ different IM-9 or IM-9/Bcl-2 cell lines were subcutaneously injected into the right dorsal flank of 6- to 8-week-old female athymic nude BALB/c mice. Following tumor growth for 7 days, the tumor-bearing mice were randomly assigned into the following two groups (10 mice per treatment group): (a) Ctrl group; (b) BetA-treated group. Mice were injected intraperitoneally for 5 consecutive days with 12 mg/kg BetA diluted in 20 mmol/l sodium citrate buffers (pH 6). Tumor volumes were evaluated according to the following formula: tumor volume (mm^3^) = 0.52 × length × width^2^. The weight of the mice was measured at 3-day intervals. At the end of the experiment, the mice were killed. Tumor net weight of each mouse was measured. Tumor tissues from control and BetA-treated mice were harvested for assay of apoptosis by terminal deoxynucleotidyl transferase–mediated dUTP nick end labeling (TUNEL) staining [75] or for assay of autophagy as measurement of the levels of LC3-II by either immunohistochemical staining or by immunoblotting using a polyclonal anti-LC3 antibody (NB600-1384) (Novus Biologicals, Littleton, CO, USA). All studies involving mice were approved by the Institutional Animal Care and Treatment Committee of Sichuan University (Chengdu, China).

### PP2A activity measurement

Cells were treated as lysed for measurement as described before [76]. Briefly, 500 μg of protein was combined with 4 μL of anti-PP2A antibody (1D6; Upstate Biotechnology) and 50 μL of protein-A-agarose beads in 500 μL. The mixture was shaken for 2 hours at 4°C, and then beads were collected by centrifugation. After 4 washes, 50 μL of phosphatase assay buffer (Upstate Biotechnology) was added to the beads, vortexed, and 50 μL of the bead slurry was added to one well of a 96-well plate. A 10mM stock of 6,8-difluoro-4-methylumbelliferyl phosphate (DiFMUP; Invitrogen) was diluted to 100μM in assay buffer, and 50 μL was added to each well. Fluorescence intensity of the product of cleavage of phosphate from DiFMUP (a synthetic phosphatase substrate) was measured using a plate reader, every 3 minutes, with shaking every 30 seconds over a 30-minute period. Specificity of the phosphatase assay for PP2A was assessed by incubating the immunoprecipitated protein with 25nM OA, a concentration that selectively for inhibits PP2A. Data presented represent the phosphate release of a sample with the background level of phosphate release of the OA-inhibited control subtracted. PP2A activity in xenografts was assayed in the same conditions. Tissue protein extraction reagent (T-PER) (Pierce Biotechnology, Rockford, IL) was used.

### Statistical analysis

The statistical analysis was performed with SPSS software (version 17.0 for Windows). Results are presented as mean ±S.D. Analysis of variance and the Tukey-Kramer multiple-comparison test were used in comparisons. *P*<0.05 was considered statistically significant.

## SUPPLEMENTARY MATERIALS FIGURES



## Abbreviations

BafA1: Bafilomycin A1
BetA: betulinic acid
DAPK: death-associated protein kinase
Cyt c: cytochrome C
MDC: monodansylcadaverine
PP2A: protein phosphatase 2A
