# Supplementary material for: PP2A mediates apoptosis or autophagic cell death in multiple myeloma cell lines
Source: Oncotarget. 2017 Aug 23;8(46):80770–89. doi: 10.18632/oncotarget.20415 (PMC5655238; doi:10.18632/oncotarget.20415)
Supplement: Supplementary file 1 [file oncotarget-08-80770-s001.pdf]

PP2A mediates apoptosis or autophagic cell death in multiple myeloma cell lines

SUPPLEMENTARY MATERIALS

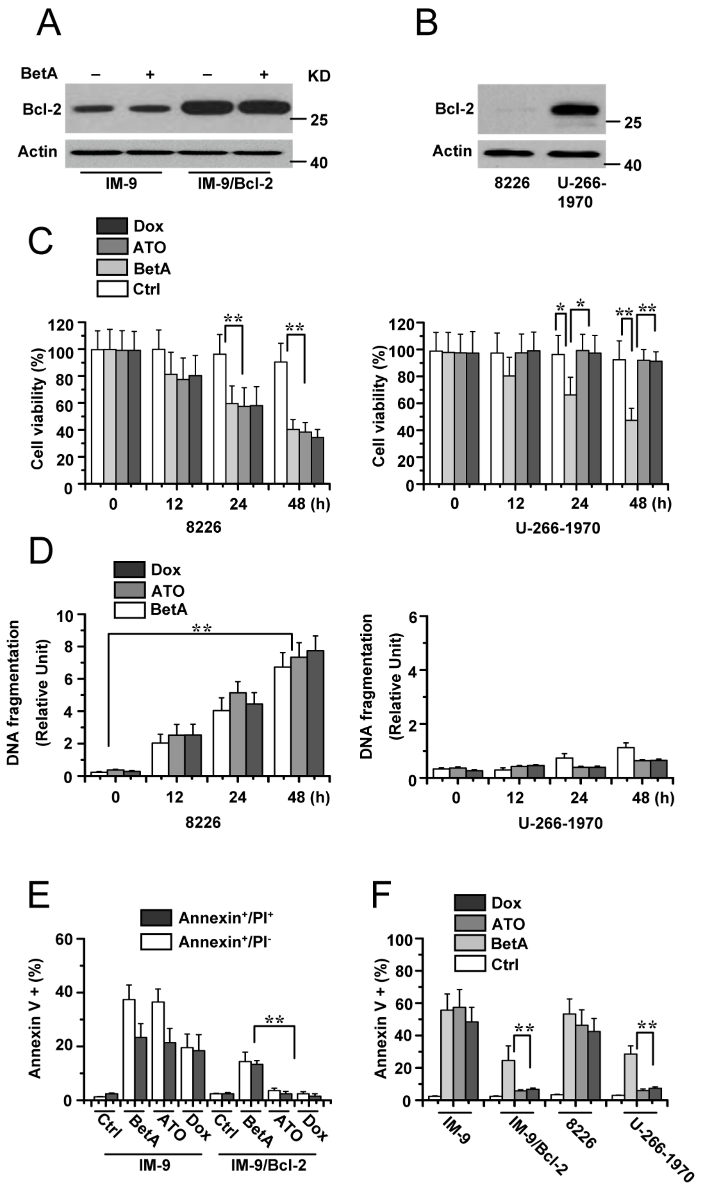

Supplementary Figure 1: (A) Bcl-2 expression in IM-9 and IM-9/Bcl-2 cells. Cells were lysed and detected for Western blot analysis. β-Actin was used as a loading control. **(B)** 8226 and U-266-1970 cells were lysed and detected for detection of Bcl-2 expression. **(C)** Indicated cells were treated as described in Figure 1B, and then cell viability was determined as described in Materials and methods. Graphs showing results of quantitative analyses (n=3, mean ± S.D. **,P<0.01). **(D)** Cells were treated as described in C. Apoptotic cell death was quantitatively detected by a cell death ELISA kit as described in Materials and methods. Graphs showing results of quantitative analyses (n=3, mean ± S.D. **,P<0.01). **(E)** Cells were treated and detected as described in Figure 1F. Cells with Annexin and PI staining were detected. Graphs showing results of quantitative analyses (n=3, mean ± S.D. **,P<0.01). **(F)** Indicated cells were treated and detected as described in Figure 1F. Graphs showing results of quantitative analyses (n=3, mean ± S.D. **,P<0.01). Representative results of three experiments with consistent results are shown.

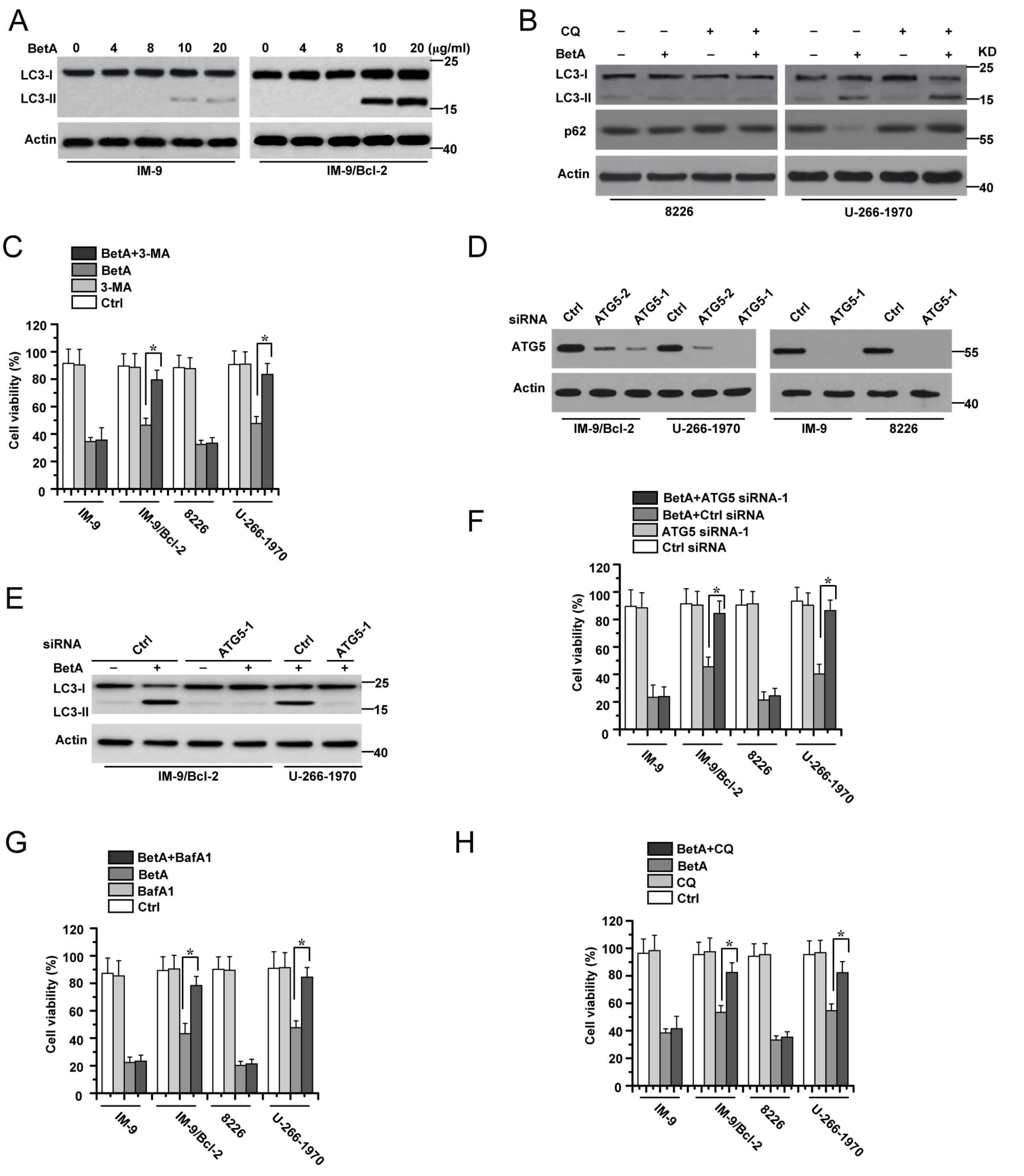

Supplementary Figure 2: (A) Indicatedcells were treated with different concentrations of BetA for 48 h, and then treated cells were collected for detecting LC3-I and LC3-II content by Western blotting, with β-Actin serving as a loading control. **(B)** Indicated cells were treated with BetA (10 μg/ml) and/or CQ (30 μM) for 48 h, and then lysed for Western blotting detection. **(C)** Cells were treated as described in Figure 2C, and then cell viability was determined as described in Materials and methods. Graphs showing results of quantitative analyses (*n*=3, mean ± S.D. *,*P*<0.05). **(D)** Cells were transfected with Ctrl, ATG5 siRNA-1 or -2 for 48 h, and then lysed and detected for Western blot analysis. **(E)** Cells were transfected with Ctrl or ATG5 siRNA-1 for 48 h, and then treated cells were collected for detecting LC3-I and LC3-II content by Western blotting. **(F-H)** Cells were treated as described in E, Figure 2E or B, and then cell viability was determined as described in Materials and methods. Graphs showing results of quantitative analyses (*n*=3, mean ± S.D. *,*P*<0.05). All data are representative of three independent experiments.

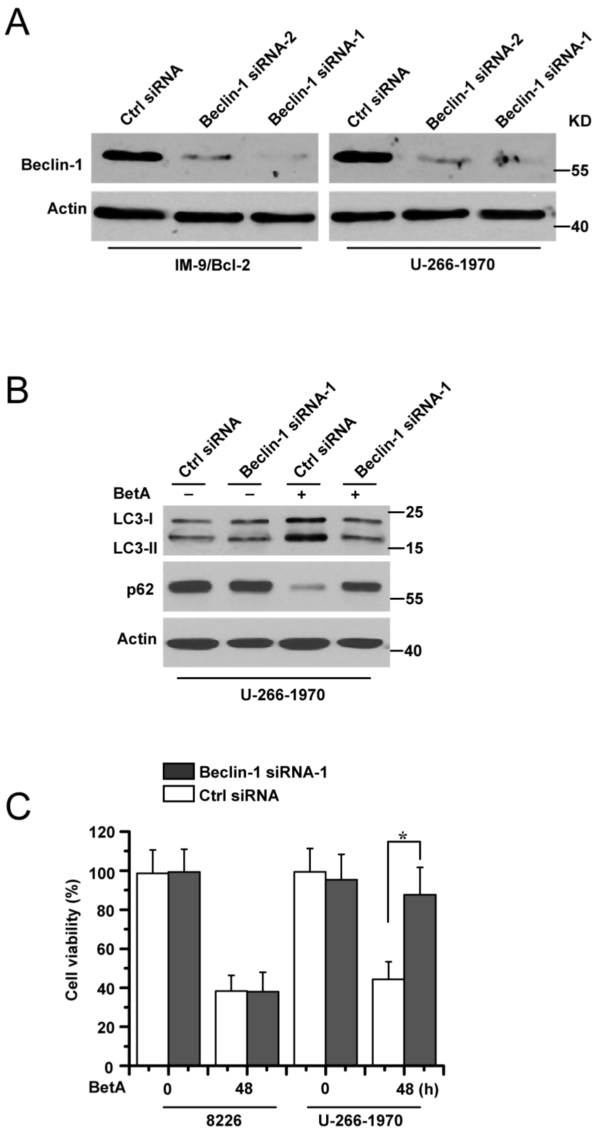

Supplementary Figure 3: (A) Cells were transfected with Ctrl, Beclin-1 siRNA-1 or -2 for 48 h, and then lysed and detected for Western blot analysis. **(B)** Cells were transfected with Ctrl or Beclin-1 siRNA-1 for 48 h, and then treated cells were collected for detecting LC3-I and LC3-II content by Western blotting. **(C)** Cells were treated as described in B, and then cell viability was determined as described in Materials and methods. Graphs showing results of quantitative analyses (n=3, mean ± S.D. *,P<0.05). Representative results of three experiments with consistent results are shown.

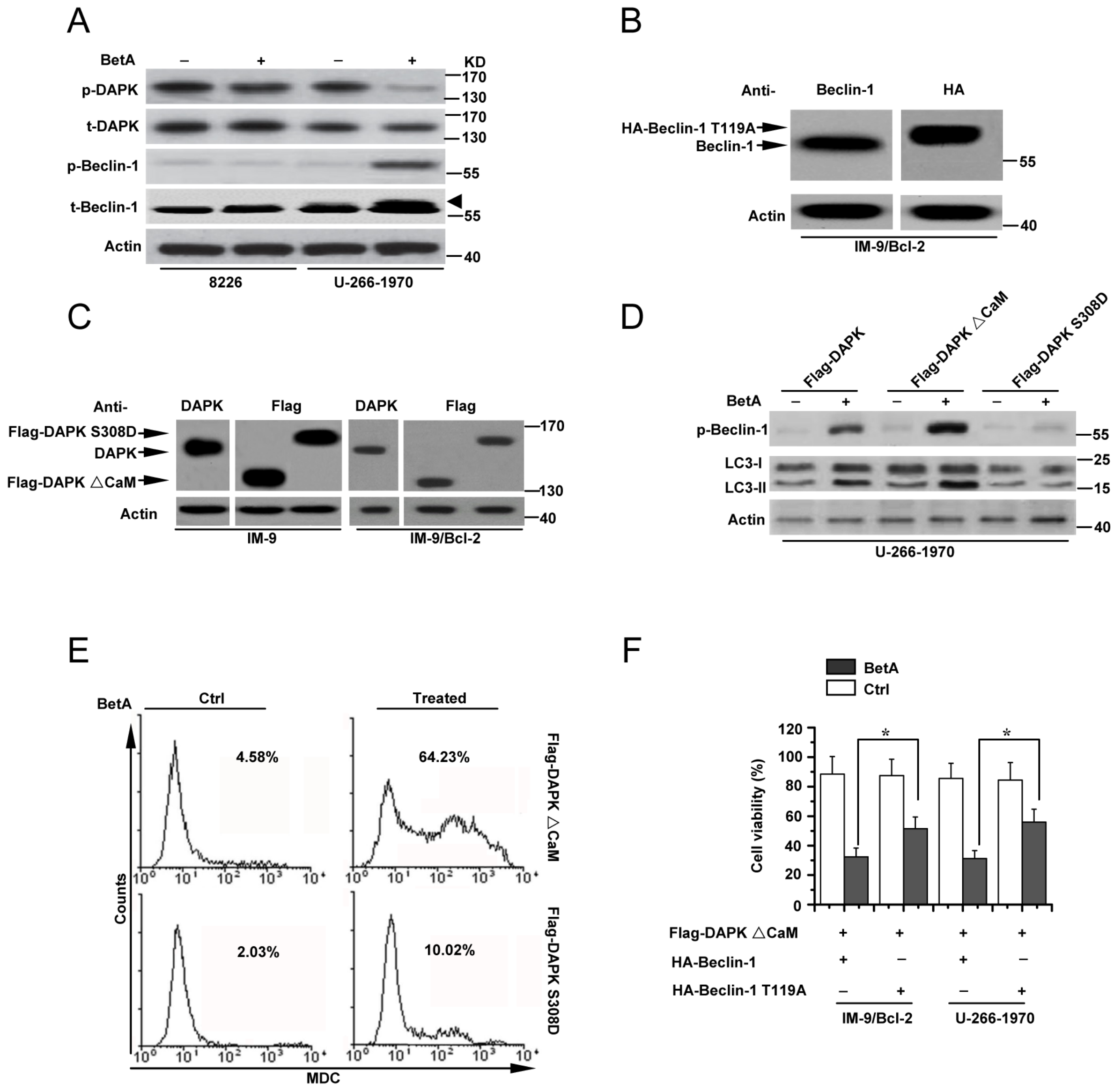

Supplementary Figure 4: (A) Indicated cells were treated with BetA for 48 h, and then lysed and detected for Western blot analysis, with β-Actin serving as a loading contro**l.** Arrowhead referred to the upshifted Beclin-1. **(B)** IM-9/ Bcl-2 cells were transfected with Ctrl vector or HA-Beclin-1 T119A for 48 h, and then cells were lysed for detection of Western blot. **(C)** IM-9 and IM-9/ Bcl-2 cells were transfected with Flag-DAPK S308D or Flag-DAPK ΔCaM for 48 h, and then cells were lysed for detection. **(D)** Indicated cells were transfected with Flag-DAPK, S308D or ΔCaM for 48 h, and then collected for detecting p-Beclin-1 and LC3 expression. **(E)** IM-9/Bcl-2 cells were transfected with Flag-DAPK S308D or Flag-DAPK ΔCaM for 48 h, and then collected for MDC staining by flow cytometry. Representative results of three experiments with consistent results are shown. **(F)** Cells were transfected with Flag-DAPK ΔCaM, HA- Beclin-1 or HA-Beclin-1 T119A for 48 h, and then treated with BetA for 48 h. Treated cells were collected for detecting cell viability. Graphs showing results of quantitative analyses (n=3, mean ± S.D. *,P<0.05). All data are representative of three independent experiments.

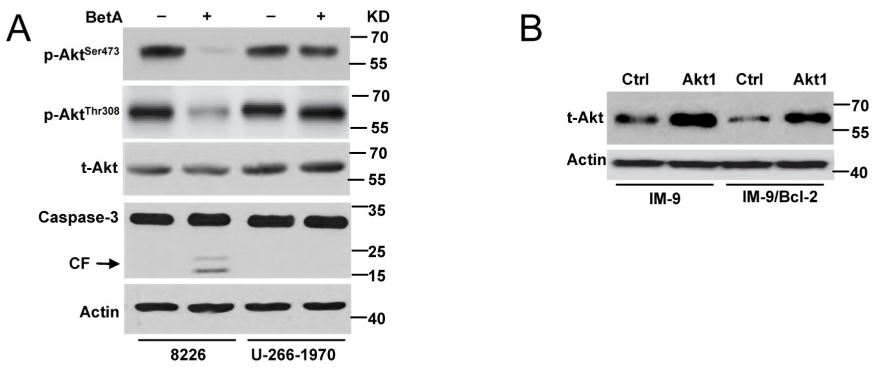

Supplementary Figure 5: (A) Indicated cells were treated with BetA for 48 h, and then lysed and detected for Western blot analysis, with β-Actin serving as a loading control. **(B)** Cells were transfected with a constitutively active Akt1 constructs for 48 h, and then lysed for Western blot detection. Representative results of three experiments with consistent results are shown.

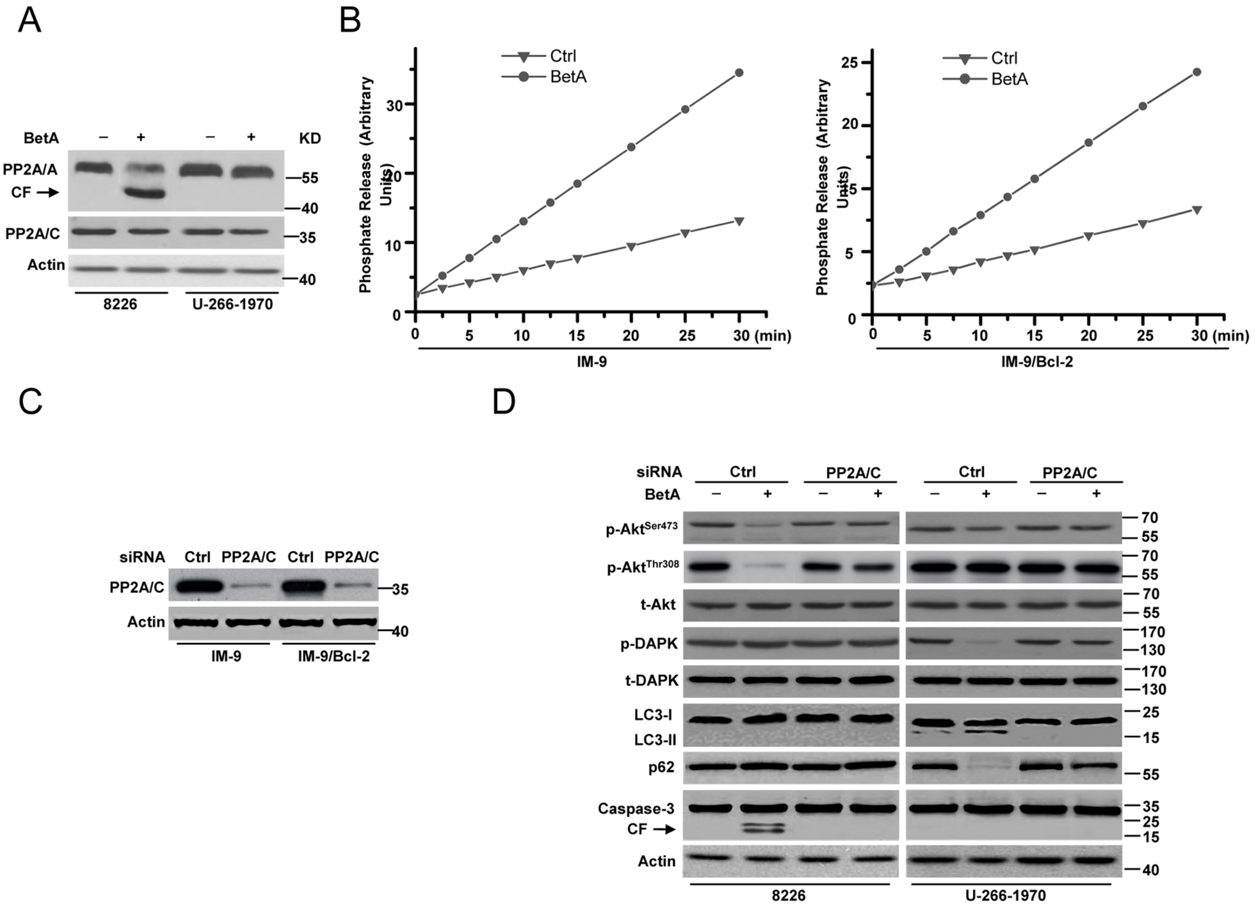

Supplementary Figure 6: (A) Indicated cells were treated with BetA for 48 h, and then lysed and detected for Western blot analysis, with β-Actin serving as a loading control. **(B)** Cell cultures or treatment with BetA followed by lysis with NP40 lysis buffer. PP2A was immunoprecipitated and assayed with the PP2A immunoprecipitation assay kit (Upstate Biotechnology), with the exception of using DiFMUP as a fluorescent substrate. The line for each sample represents the phosphate release from a given sample after subtraction of the OA-inhibited control reactions ($P<0.05$). **(C)** Cells were transfected with PP2A/C or Ctrl siRNA for 48 h, and then collected for detecting PPA/C expression by Western blotting. **(D)** Cells were transfected with PP2A/C or Ctrl siRNA for 48 h, and then treated with BetA for 48 h. Treated cells were lysed for Western blot detection. All data are representative of three independent experiments.
